# Effects of exergaming on brain health in aging: a scoping review of current evidence

**DOI:** 10.3389/fpubh.2026.1912065

**Published:** 2026-08-10

**Authors:** Shun Liang, Jun Yin

**Affiliations:** School of Physical Education and Training, Capital University of Physical Education and Sports, Beijing, China

**Keywords:** brain-derived neurotrophic factor, cognitive function, electroencephalography, exergaming, neuroimaging, neuroplasticity, older adults

## Abstract

**Background:**

The rapid aging of the global population has increased the need for engaging, scalable interventions to support brain health in aging populations. Exergaming, which integrates physical activity with game-based cognitive demands, has shown promise in this regard.

**Objective:**

This scoping review aimed to map and synthesize current evidence on electrophysiological, functional, structural, and molecular (BDNF) markers associated with exergaming in older adults, and to identify whether existing comparative studies suggest benefits beyond or comparable to traditional exercise.

**Methods:**

This review was conducted as a scoping review and reported in accordance with the PRISMA extension for Scoping Reviews (PRISMA-ScR). PubMed, Scopus, Web of Science, and PsycINFO were searched from inception to October 20, 2025. Two independent reviewers screened records and charted data, with disagreements resolved by a third reviewer.

**Results:**

We included 22 studies (17 randomized controlled trials and 5 non-randomized controlled trials). Electrophysiologically, eight studies reported changes in event-related potential components and theta/alpha rhythm power. Functionally, several studies reported reduced task-related prefrontal activation or altered resting-state connectivity, although these changes may reflect neural efficiency, motor automaticity, task familiarization, or a combination of these processes. Structurally, two of three MRI studies reported increased gray matter volume or attenuated volume decline in selected regions. At the molecular level, seven studies measured peripheral BDNF, but between-group superiority over traditional exercise was inconsistent.

**Conclusion:**

Current evidence suggests that exergaming may be associated with favorable changes in several markers of brain health in older adults, including neuroelectrical activity, task-related and resting-state brain function, selected structural indices, and peripheral BDNF. However, the evidence remains preliminary because studies differ substantially in population, intervention content, dose, comparator, and outcome measurement. Claims about cognitive-motor synergy and causal pathways linking BDNF, brain structure, functional efficiency, and cognition should therefore be interpreted as hypotheses for future research rather than established conclusions. Comparable effects should be interpreted as showing that game-based delivery can preserve exercise-related benefits, not as proof that gaming itself adds a distinct neurobiological effect. Overall, the current evidence more strongly supports comparability and potential implementation value than a distinct neurobiological advantage of gaming.

## 1 Introduction

The rapid growth of the global aging population is profoundly reshaping public health and socioeconomic landscapes. The latest estimates from the World Health Organization (WHO) indicate that by 2030, one in six people worldwide will be aged 60 years or over. In the WHO framework, brain health refers to brain functioning across cognitive, sensory, social–emotional, behavioral, and motor domains across the life course (1). This definition is particularly relevant to older adults because aging-related changes occur across neuroanatomical, neurophysiological, and neurofunctional levels, and these changes may contribute to impairments in cognition, mobility, and everyday functioning (1).

Traditional unimodal exercise interventions, such as aerobic, resistance, or balance training alone, can benefit cognition in later life, but their effects may vary according to how closely the intervention engages cognitive, perceptual, and motor-control demands (2, 3). Exergaming, commonly understood as technology-supported gameplay that requires bodily movement, may provide a structured context in which physical activity is coupled with attention, visuospatial processing, rapid decision-making, working-memory updating, and feedback-based learning (4). Therefore, exergaming is best viewed as a form of integrated motor-cognitive training rather than as a simple addition of a game layer to exercise. Because exercise alone can influence brain-related outcomes, any proposed contribution of game elements must be evaluated as an incremental effect beyond adequately matched physical activity.

The theoretical rationale for exergaming is that simultaneous motor and cognitive engagement may create enriched training demands that are relevant to neuroplastic adaptation (4–6). However, whether this integration produces a true synergistic effect beyond matched physical exercise remains uncertain because many available studies compare exergaming with usual care or a single active control rather than factorial designs that isolate the cognitive-game component. In this review, we therefore use the term cognitive-motor coupling as a mechanistic hypothesis, and we distinguish this hypothesis from direct evidence of superiority over traditional exercise.

Accordingly, the primary objective of this scoping review was to map the available evidence on exergaming and multidimensional markers of brain health in older adults. Specifically, we organized outcomes into molecular, structural, functional, neuroelectrical, and behavioral levels, and we examined whether current studies provide evidence of comparable or superior effects relative to traditional exercise. We further distinguished comparable effects from incremental benefit, because equivalence to exercise alone does not establish a unique gaming-specific neurobiological mechanism.

## 2 Methods

### 2.1 Search strategy

This review was designed as a scoping review because the field includes heterogeneous populations, intervention types, comparators, study designs, and brain-related outcomes. The review was guided by established scoping review methodology and reported according to PRISMA-ScR (7–10). Four electronic databases were searched: PubMed, Scopus, Web of Science, and PsycINFO. Searches covered all records available up to October 20, 2025. The search combined controlled vocabulary where available, such as MeSH terms in PubMed, with free-text terms related to exergaming/active video games/virtual reality games, older adults, and brain-related outcomes. The PubMed search strategy is summarized in Table 1, and the complete database-specific search strings are provided in Supplementary Table S1.

**Table 1:** Search strategy summary.

Electronic database	Search strategy/Reporting
PubMed, Scopus, Web of Science, PsycINFO	Searches combined controlled vocabulary where available and free-text terms for older adults, exergaming/active video gaming/virtual reality games, and brain-related outcomes. PubMed example: ((exergam* OR “active video game*” OR “virtual reality” OR “serious game*” OR “video game*” OR “computer game*” OR “virtual cycling” OR “Wii” OR “Kinect”) AND (older adult* OR elderly OR aging OR ageing OR geriatric*) AND (brain OR cognition OR EEG OR ERP OR fNIRS OR fMRI OR MRI OR BDNF OR “brain-derived neurotrophic factor”)). Database-specific syntax was adapted for Scopus, Web of Science, and PsycINFO.

### 2.2 Inclusion criteria

Eligibility criteria followed the Population-Concept-Context (PCC) framework recommended for scoping reviews. Population: studies involving older adults or clinical older populations were included. Concept: interventions were included if they met the criteria for exergaming or active video gaming, namely technology-supported gameplay requiring active bodily movement beyond simple finger movement. Eligible systems included commercial platforms such as Nintendo Wii, Xbox Kinect, and dance-mat systems, as well as custom-developed virtual cycling, balance, stepping, boxing, or sport-related games. Context: no restriction was placed on care setting or degree of immersion. Comparators included usual care, no intervention, conventional exercise, balance training, resistance/stretching exercise, or motor-cognitive dual-task training. Outcomes included brain-related measures obtained through EEG/ERP, fNIRS, fMRI, MRI, or peripheral BDNF assessment, and cognitive outcomes were charted when reported alongside these markers. Studies were excluded if they used only gamepad-controlled sedentary games, non-exercise VR exposure, cognitive or board games without physical activity, protocols without empirical outcome data, conference abstracts, reviews, meta-analyses, posters, and non-English publications.

Evidence sources were included only if they met all eligibility criteria described above. Reviews, meta-analyses, protocols, conference abstracts, posters, non-empirical publications, and non-English publications were excluded.

### 2.3 Study selection

**Figure 1:**
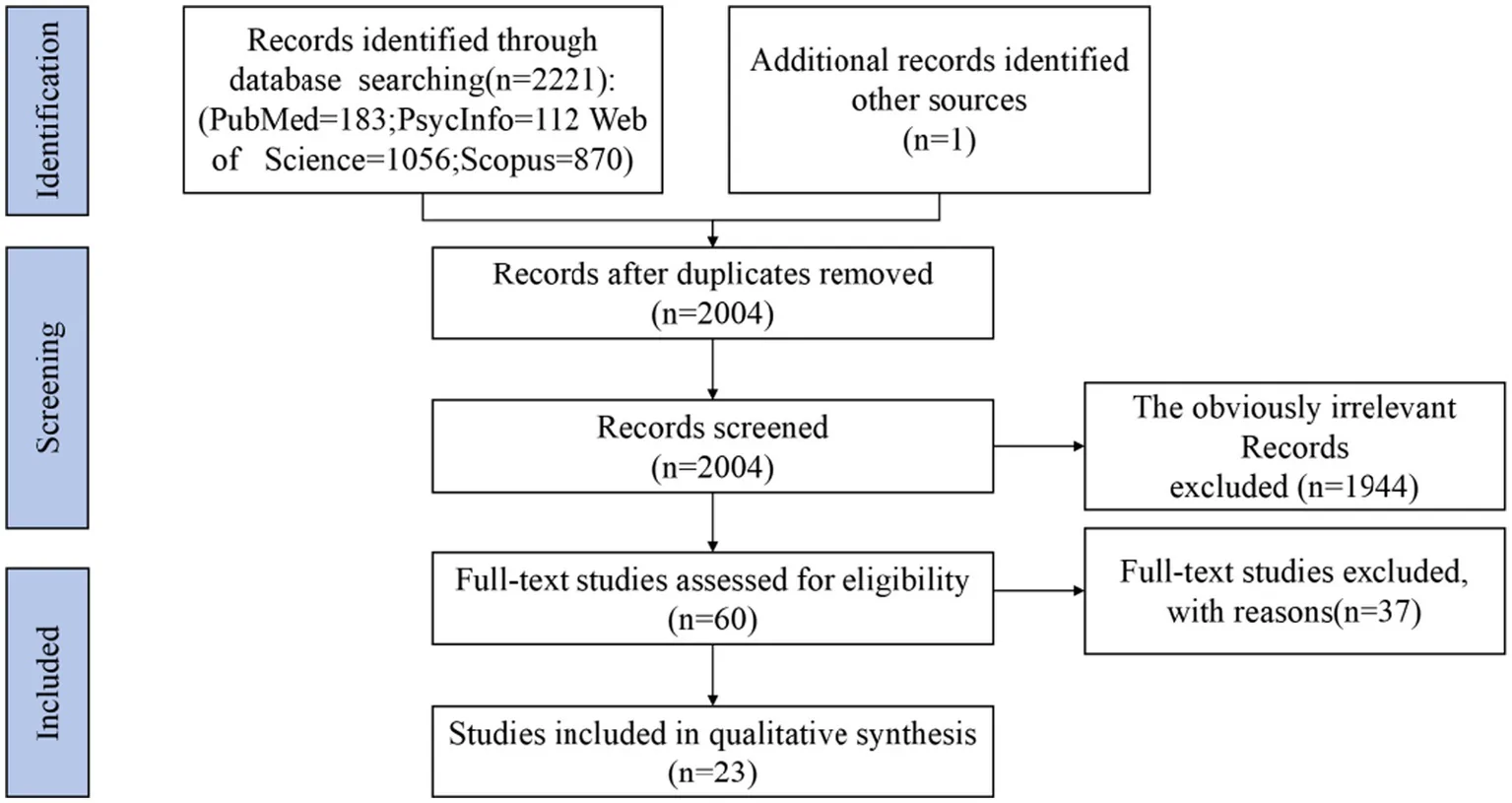
PRISMA flowchart.

Following the electronic database searches, two researchers independently screened the search results using a three-step process. First, duplicate records were removed using reference management software (Endnote 20). Second, titles and abstracts were assessed against the eligibility criteria. If a decision on eligibility could not be made based on the information in the title and abstract alone, the third step involved full-text screening. In cases of disagreement between researchers regarding study eligibility, a third researcher was consulted to reach a final decision. The process of identification, screening, and selection is illustrated in Figure 1 and described in more detail in Section 3.1.

### 2.4 Data extraction

The following data were extracted into a standardized data sheet: publication (author/year), participant characteristics (sample, sample size, age), intervention method for the experimental group, intervention method for the control group, intervention protocol (intensity, duration, frequency, session time), and outcomes (cognitive tasks, brain imaging tools, measurement tools, neural factors). If studies reported results at different time points, the post-intervention assessment results from the final time point were extracted. Data were extracted independently by two reviewers, and any discrepancies were resolved through discussion with a third reviewer.

Because the objective was to map the range, characteristics, and knowledge gaps of existing evidence rather than to estimate a pooled effect size, no formal risk-of-bias assessment was performed. This decision is consistent with scoping review methodology, but the absence of critical appraisal was considered when interpreting the strength of the conclusions and is acknowledged in the limitations.

To evaluate the effects of exergaming on multidimensional indicators of brain health in older adults, data were synthesized descriptively rather than meta-analytically. First, outcomes were charted by biological level: molecular markers, brain structure, brain function, neuroelectrical activity, and behavioral cognition. Second, studies within each level were categorized by effect direction and comparator type. Third, we distinguished evidence showing superiority over traditional exercise from evidence showing comparable effects or within-group change only. Fourth, when findings were heterogeneous, we examined potential sources of variability, including population, exercise modality, cognitive demand, immersion level, intervention dose, exercise intensity, comparator, and measurement timing. Evidence of comparability was interpreted conservatively as maintenance of exercise-related benefit rather than proof of an added gaming-specific effect.

## 3 Results

### 3.1 Study selection

The complete study selection process is illustrated in Figure 1. A total of 2,221 records were identified. After removing duplicates, 2,004 records remained. Following title, abstract, and full-text screening, 22 studies met the inclusion criteria. Based on their outcome measures, the included studies were categorized into four sub-groups: (1) neuroelectrical activity, (2) brain function, (3) brain structure, and (4) brain-derived neurotrophic factors. The final set included 17 randomized controlled trials and 5 non-randomized controlled trials.

### 3.2 Study selection and characteristics

Fifteen studies involved cognitively healthy older adults, three involved individuals with dementia or mild cognitive impairment (MCI), one involved older adults with type 2 diabetes, and three involved individuals with Parkinson’s disease (PD). All studies that clearly identified the platform or game type were classified as non-immersive exergames. In these studies, game visuals were presented via televisions, projectors, or computer screens, with users remaining in a real-world environment. No study used head-mounted displays (e.g., Oculus Rift, HTC Vive) to deliver a fully immersive experience. Even studies described as virtual reality cycling (e.g., those by the Anderson-Hanley team (11, 12)) used large screens to display virtual scenarios and were therefore classified as non-immersive or semi-immersive virtual reality. Regarding commercial versus custom systems, both types were represented. Commercial platforms mainly comprised off-the-shelf devices such as Xbox 360 Kinect (13–15) and the Dance Dance Revolution dance mat (16). Custom-built systems included the “Puzzle & Fox” and step/balance games developed by Mueller’s team, as well as the virtual bicycle Exer-tour/Exer-score used by the Anderson-Hanley team (11, 12). Notably, 12 of the 22 studies did not provide sufficient platform or game details to determine their level of immersion or commercial status. This incomplete reporting indicates that future studies should provide detailed information on platform name, game type, degree of immersion, and whether the system was commercial or custom developed. Control group interventions included usual care, traditional aerobic exercise, balance training, resistance and stretching exercises, and motor-cognitive dual-task training. Seven studies reported exercise intensity. All studies reported the intervention protocol, including duration, frequency, and session length. Six studies employed common cognitive tasks during brain imaging (Flanker test, attention task, eye-closing task, emotional memory task, and motor imagery). Two studies used the exergame itself as the cognitive task during imaging. Three studies used walking as the imaging task, whereas the remaining studies did not use a synchronized task during imaging. Four studies measured event-related potentials (ERP), four reported electroencephalography (EEG) findings, four used magnetic resonance imaging (MRI), including two that used functional MRI (fMRI), and four used functional near-infrared spectroscopy (fNIRS). Seven studies measured BDNF levels, all using enzyme-linked immunosorbent assay (ELISA) kits. Specific details are presented in Tables 2, 3.

**Table 2:** Study design and participant characteristics of included studies.

Author, year	Study design	Participants	Group	Age
Julia Dimitrova (2016) (18)	Controlled experiment	Older adults	EG:29	EG:70.7 ± 5.4
Wu (2023) (17)	RCT	Adults with mild-to-moderate dementia	EG:13 CG:11	Age≥65 years
Alexandra Schättin (2016) (19)	RCT	Older adults	EG:13 CG:13	EG: 73–83 CG:72.25–81.75
Gholamreza Olyaei 2022 (20)	RCT	Older adults	EG:25 CG:27	EG: 71.22 ± 5.82 CG:70.43 ± 5.24
Helen Müller (2025) (21)	Before-after study	Older adults	21	74.80 ± 0.81
Helen Müller (2023) (22)	Cross-sectional study	Older adults	28	74.57 ± 0.78
Imran Amjad (2019) (13)	RCT	MCI	EG:18 CG:20	Not reported
Lan-Ya Chuang (2015) (16)	RCT	Older adults	EG:8 CG1:7 CG2:11	EG:69.43 ± 3.82 CG1:67.01 ± 1.67 CG2:68.25 ± 3.96
Eva Schaeffer, 2022 (28)	Controlled experiment	EG: PD; CG: healthy older adults	EG:17 CG:16	EG:58 ± 10 CG:58 ± 12
Manuela Adcock (2019) (29)	RCT	Older adults	EG:15 CG:16	EG:77.0 ± 6.4 CG:70.9 ± 5.0
Cay Anderson-Hanley (2018) (11)	RCT	MCI	EG1:7 EG2:7	EG1:80.9 ± 12.3 EG:75.4 ± 9.83
Jadwiga Kubica (2019) (23)	RCT	Older adults	EG:15 CG1:28 CG2:23	EG:66.32 ± 0.60 CG1:65.27 ± 0.85 CG2:64.39 ± 0.99
Lanxin Ji (2017) (26)	RCT	Older adults	EG:12 CG:12	EG:67.0 ± 6.40 CG:73.0 ± 8.00
Pei-Ling Wong (2024) (24)	RCT	PD	EG:11 CG1:11 CG2:10	EG1:65.86 ± 5.60 CG1: 67.63 ± 6.92 CG2:64.94 ± 4.98 CG167.63 ± 6.92 CG2:64.94 ± 4.98
Inbal Maidan (2018) (27)	RCT	PD	EG:30 CG:34	EG:70.1 ± 1.3 CG:73.1 ± 1.1
Ying-Yi Liao (2021) (14)	RCT	Older adults	EG:23 CG:23	EG:79.6 ± 9.0 CG:83.8 ± 5.1
Patrick Eggenberger (2016) (25)	RCT	Older adults	EG: 19 CG:14	EG:72.8 ± 5.9 CG:77.8 ± 7.4
Nadia Rizki Rahmawati (2019) (30)	RCT	Older women	EG:15 CG:15	EG:69.5 ± 5.77 CG:71.2 ± 7.32
Monteblanco Cavalcante (2021) (15)	Before-after study	Older adults	9	71.9 ± 5.7
Christian Brinkmann (2017) (32)	Cross-over comparison	Older men with type 2 diabetes	30	71 ± 4
Cay Anderson-Hanley (2012) (12)	RCT	Older adults	EG1:38 EG2:41	EG1:75.7 ± 9.9 EG:81.6 ± 6.2

**Table 3:** Intervention and outcome-measurement characteristics of included studies.

Author, year	Experimental intervention	Control intervention	Intervention time	Outcome task and measurement	Neurotrophic factor
Julia Dimitrova (2016) (18)	Exergaming (Cycling)	Physical exercise	Exercise intensity: Moderate Period: 1 time Duration: 20 min/time Period: 1 times Duration: 20 min/ time	Cognitive task: Stroop task Brain imaging/tool: ERP	–
Wu, 2023 (17)	Exergaming (Cycling)	Aerobic exercise (Cycling)	Exercise intensity: NA Period: 4 weeks Frequency: 3 times/week Duration: 30–40 min/time Period: 4 weeks Frequency:3times/week Duration:30–40 min/time	Cognitive task: Eriksen Flanker Test Brain imaging/tool: ERP Measurement tool: -	–
Alexandra Schättin, 2016 (19)	Exergaming (Balance)	Balance exercise	Exercise intensity: Moderate to high Period: 24 times Duration: 30 min/time Period: 24 times Duration: 30 min/ time	Cognitive task: Visual attention task Brain imaging/tool: ERP Measurement tool: -	–
Gholamreza Olyaei 2022 (20)	Exergaming (Wii Fit)	Motor-cognitive dual-task	Exercise intensity: Moderate to high Period: 16 times Frequency: 3 times/week Duration: 60 min/time Period: 16 times Frequency:3times/week Duration: 60 min/ time	Cognitive task: - Brain imaging/tool: EEG Measurement tool: -	–
Helen Müller (2025) (21)	Exergaming	NA	Exercise intensity: NA Period: 4 weeks Frequency: 3 times/week Duration: 45 min/time Period: 4 weeks Frequency:3times/week Duration: 45 min/ time	Cognitive task: Exergaming Brain imaging/tool: EEG Measurement tool: -	–
Helen Müller (2023) (22)	Exergaming	NA	Exercise intensity: NA Period: 1 time Duration: 45 min/time Period: 1 time Duration: 45 min/ time	Cognitive task: Exergaming Brain imaging/tool: EEG Measurement tool: -	–
Imran Amjad (2019) (13)	Exergaming (Xbox 360 Kinect)	Range of motion exercises	Exercise intensity: NA Period: 6 weeks Frequency: 5 times/week Duration: 25–30 min/ time Period: 6 weeks Frequency:5times/week Duration: 25–30 min/ time	Cognitive task: Eye-closing task Brain imaging/tool: EEG Measurement tool: -	–
Lan-Ya Chuang (2015) (16)	Exergaming (dance)	CG1: Brisk walking CG2: Blank group	Exercise intensity: Moderate to high Period: 12 weeks Frequency: 3 times/week Duration: 30 min/time Period: 12 weeks Frequency:3times/week Duration: 30 min/ time	Cognitive task: Flanker task Brain imaging/tool: ERP Measurement tool: -	–
Eva Schaeffer (2022) (28)	Exergaming (Microsoft Corporation)	Exergaming (Microsoft Corporation)	Exercise intensity: Moderate Period: 6 weeks Frequency: 3 times/week Duration: 45 min/time Period: 6 weeks Frequency:3times/week Duration: 45 min/ time	Cognitive task: - Brain imaging/tool: MRI Measurement tool: BDNF	ELISA
Manuela Adcock (2019) (29)	Exergaming	Usual care	Exercise intensity: NA Period: 48 times Frequency: 3 times/week Duration: 30–40 min/ time Period: 48 times Frequency:3times/week Duration: 30–40 min/ time	Cognitive task: - Brain imaging/tool: MRI Measurement tool: -	–
Cay Anderson-Hanley (2018) (11)	EG1: Exergaming(High cognitive) EG2: Exergaming(Low cognitive)	NA	Exercise intensity: NA Period: 6 months Frequency:2-4times/week Duration: 20–45 min/ time Period: 6 months Frequency:2-4times/week Duration: 20–45 min/ time	Cognitive task: - Brain imaging/tool: MRI Measurement tool: BDNF	ELISA
Jadwiga Kubica (2019) (23)	Exergaming (balance)	CG1:balance exercise CG2: Usual care	Exercise intensity: Moderate Period: 12 times Frequency: 3 times/week Duration: 30–60 min/ time Period: 12 times Frequency:3times/week Duration: 30–60 min/ time	Cognitive task: - Brain imaging/tool: fMRI Measurement tool: BDNF	ELISA
Lanxin Ji (2017) (26)	Exergaming (Wii fit)	Usual care	Exercise intensity: NA Period: 6 weeks Frequency: 7 times/week Duration: 30 min/time Period: 6 weeks Frequency:7times/week Duration: 30 min/ time	Cognitive task: Emotional memory task Brain imaging/tool: fMRI Measurement tool: -	–
Pei-Ling Wong (2024) (24)	Exergaming	CG1: Cognitive-motor CG2: Usual care	Exercise intensity: NA Period: 12 times Duration: 40 min/ time Period: 12 times Duration: 40 min/ time	Cognitive task: Obstacle walking tasks Brain imaging/tool: fNIRS Measurement tool: -	–
Inbal Maidan (2018) (27)	Exergaming (Cycling)	Aerobic exercise (Cycling)	Exercise intensity: NA Period: 6 weeks Frequency: 3 times/week Duration: 45 min/time Period: 6 weeks Frequency:3times/week Duration: 45 min/ time	Cognitive task: Different walking tasks Brain imaging/tool: fNIRS Measurement tool: -	–
Ying-Yi Liao (2021) (14)	Exergaming	Combined physical exercise	Exercise intensity: Moderate Period: 12 weeks Frequency: 3 times/week Duration: 60 min/time Period: 12 weeks Frequency:3times/week Duration: 60 min/ time	Cognitive task: MoCA tasks Brain imaging/tool: fNIRS Measurement tool: -	–
Patrick Eggenberger (2016) (25)	Exergaming (Dance)	Balance exercise	Exercise intensity: NA Period: 8 weeks Frequency: 3 times/week Duration: 30 min/time Period: 8 weeks Frequency:3times/week Duration: 30 min/ time	Cognitive task: Walking tasks Brain imaging/tool: fNIRS Measurement tool: -	–
Nadia Rizki Rahmawati (2019) (30)	Exergaming (boxing) + light intensity aerobic exercise	Light intensity aerobic exercise	Exercise intensity: NA Exergaming Period: 8 weeks Frequency: 3 times/week Duration: 25–30 min/ time light intensity aerobic exercise Period: 8 weeks Frequency: 5 times/week Duration: 10–15 min/ time Exergaming Period: 8 weeksFrequency:3times/week Duration: 25–30 min/ time light intensity aerobic exercise Period: 8 weeks Frequency:5times/week Duration: 10–15 min/ time	Cognitive task: - Brain imaging/tool: - Measurement tool: BDNF	ELISA
Monteblanco Cavalcante (2021) (15)	Exergaming	functional balance training	Exercise intensity: NA Period: 6 weeks Frequency:2times/week Duration: 40 min/ time Period: 6 weeks Frequency:2times/week Duration: 40 min/ time	Cognitive task: - Brain imaging/tool: - Measurement tool: BDNF	ELISA
Christian Brinkmann (2017) (32)	Exergaming (Cycling)	Aerobic exercise (Cycling)	Period: 1 time Duration: 30 min/time Duration: 30 min/ time	Cognitive task: - Brain imaging/tool: - Measurement tool: BDNF	ELISA
Cay Anderson-Hanley (2012) (12)	Exergaming (Cycling)	Aerobic exercise (Cycling)	Exercise intensity: Moderate Period: 3 months Frequency: 5 times/week Duration: 45 min/time Period: 3 months Frequency:5times/week Duration: 45 min/ time	Cognitive task: - Brain imaging/tool: - Measurement tool: BDNF	ELISA

EG, Experimental Group; CG, Control Group; MCI, Mild cognitive impairment; PD, Parkinson’s disease; ELISA, Enzyme-linked immunosorbent assay; RCT, Randomized controlled trial, BDNF: Brain-derived neurotrophic factor; MI, Motor imagery; AO, Action observation; AOMI, Action observation combined with motor imagery.

^*^Although the study by Amjad (13) mentioned targeting older adults in the title, the full text did not provide the participants’ mean age, standard deviation, or inclusion age range.

For readability, the characteristics of included studies are presented in two linked tables: Table 2 summarizes study design and participant characteristics, whereas Table 3 summarizes intervention and outcome-measurement characteristics.

### 3.3 Main findings

The main findings on exergaming interventions and older adults’ brain health are presented below, organized according to brain imaging modalities and BDNF changes.

(1) Exergaming and Neuroelectrical Activity

Eight studies reported on the relationship between exergaming interventions and neuroelectrical activity in older adults.

Two ERP studies reported that exergaming interventions improved neurophysiological measures, as reflected by increased amplitudes and shortened latencies of cognitive-related components (16, 17). One study found significant changes favoring exergaming over traditional aerobic exercise, whereas the other found no significant between-group differences in neuroelectrical activity. A study comparing 12-week exergaming with traditional aerobic exercise in a dementia cohort found that exergaming significantly increased N2 amplitude and decreased N2 latency in frontal and parietal regions. In addition, P3b amplitude in the frontoparietal regions increased for both congruent and incongruent trials of the Eriksen Flanker Test, suggesting that exergaming had a greater effect on selective attention and working memory than traditional aerobic exercise (17). Another study involved 26 sedentary, healthy older women assigned to an exergaming group, a brisk walking group, or a control group. The exergaming and walking groups performed moderate-intensity exercise three times per week for 3 months, whereas the control group maintained a sedentary lifestyle. All participants performed the Flanker task before and after the intervention. Both exergaming and brisk walking shortened N2 and P3 latencies compared with the control condition, suggesting that exergaming was as effective as brisk walking in improving inhibitory control in older adults (16). This pattern supports comparability rather than gaming-specific superiority unless exercise dose, cognitive challenge, and motivational exposure are explicitly matched and separated.

Conversely, three studies found that exergaming interventions were associated with lower power in specific frequency bands across different brain regions (18–20). One study comparing a single session of exergaming with exercise without added cognitive components found that both groups showed a significant increase in EEG amplitude at the Cz electrode between 320 and 700 ms after stimulus presentation during a Stroop task (18). During an attention task, a study comparing 24 sessions of balance-type exergaming with traditional balance exercise in healthy older adults found that the exergaming group exhibited lower relative theta power in the prefrontal region (19). Another study comparing exergaming with motor-cognitive dual-task training found that both groups showed reductions in late contingent negative variation (CNV) and alpha/beta event-related desynchronization (ERD) at Cz and C3 channels after training (20).

Three EEG studies found differential effects of exergaming interventions on power across frequency bands (13, 21, 22). One study involving individuals with MCI assigned to either exergaming or range-of-motion training found that a single session of exergaming significantly increased delta and theta power. After 6 weeks of intervention, alpha, delta, and theta power further increased in the exergaming group, whereas no significant changes were observed in the range-of-motion group (13). Another study conducted 12 exergaming sessions with 21 older adults and collected EEG data at six time points. Frontal theta power increased progressively during exergaming sessions, in parallel with gradual behavioral performance improvement (21). A further study found that exergaming significantly increased frontal theta power compared with training in a non-gaming scenario. During exergaming, right parietal alpha power spectral density increased from no-game to low-difficulty and high-difficulty game conditions, whereas left parietal alpha power decreased. In the difficult exergaming condition, increased right-hemisphere alpha power was observed only under high-difficulty gaming. These findings suggest that EEG responses may depend on task-specific demands across game modes (22).

(2) Exergaming and Brain Function

Six studies investigated the relationship between exergaming interventions and brain function in older adults.

Three of these studies observed no significant between-group differences in brain function metrics (23–25). One study comparing exergaming with a combined motor-cognitive intervention found no significant between-group differences in brain activation during a complex task, although both intervention groups showed greater improvement in behavioral cognitive tests (24). Another study reported that both exergaming and traditional moderate-intensity balance training modulated activation in sensorimotor cortices involved in postural control. Under action observation (AO) and action observation combined with motor imagery (AOMI) conditions, activation increased in sensory cortices related to motion perception, suggesting visual system involvement in automatic postural control in older adults (23). A study comparing 36 sessions of exergaming with conventional physical exercise showed decreased prefrontal activation during a cognitive task in both groups (25). These null between-group findings show that exergaming can produce similar functional changes to active comparators, but they do not isolate an added effect of the game component.

The other three studies reported decreased task-related activation after intervention, with two suggesting that this trend might be more pronounced following exergaming (14, 26, 27). In PD patients, both 6-week exergaming cycling and regular cycling reduced prefrontal activation during walking. The exergaming group showed a greater reduction in activation, particularly in the right prefrontal region during dual-task and obstacle-walking conditions, suggesting enhanced gait automaticity and reduced cognitive reliance (27). A study comparing exergaming with balance training showed that both interventions significantly decreased bilateral prefrontal oxygenation during fast walking. However, the exergaming group demonstrated a more pronounced decrease in left prefrontal oxygenation at the end of a 30-s walk test, and this change was correlated with improved executive function (14). A resting-state fMRI study found that 6 weeks of daily 30-min exergaming altered regional homogeneity (ReHo), decreasing ReHo in the posterior cingulate cortex and increasing ReHo in the cingulate gyrus and temporal, parietal, and occipital lobes. Changes in striatal-thalamic connectivity were also correlated with improvements in executive function (26). Even when exergaming appears more favorable, such differences should be interpreted cautiously unless the comparator controls for aerobic load, motor complexity, feedback, and engagement.

(3) Exergaming and Brain Structure

Three MRI-based studies investigating the effects of exergaming on brain morphology in older adults were included (26, 28, 29). Two studies observed volume increases in specific brain regions following exergaming (26, 28). A 6-week intervention study in older adults with PD found that exergaming significantly increased volume in the left hippocampal subfields CA1, CA4/dentate gyrus, and subiculum, with a more pronounced increase in dentate gyrus volume in PD patients than in healthy older adults (28). Another study using Nintendo Wii found that the intervention group showed increased gray matter volume in frontal and posterior cingulate regions, while striatal volume remained stable. In contrast, the control group showed a decline in striatal volume (26). One study found no significant increase in whole-brain or regional volumes after 48 sessions of 30–40 min of exergaming compared with usual care, despite clear improvements in executive function (29). Because most structural studies did not include dose-matched exercise and non-game cognitive controls, they cannot determine whether structural changes reflect game-specific effects.

(4) Exergaming and BDNF

Seven studies investigated the effect of exergaming interventions on BDNF levels in older adults.

Three studies reported BDNF changes favoring exergaming or exergaming plus exercise over comparator conditions (15, 28, 30). A 6-week intervention found that exergaming increased serum BDNF levels in both PD patients and healthy older adults (28). Another study observed increased salivary BDNF after 12 sessions of Xbox 360 Kinect training (15). A study in older women found that boxing exergaming combined with light aerobic exercise produced larger improvements in cognitive performance and BDNF than light aerobic exercise alone (30). However, this comparison may also reflect differences in intervention intensity, novelty, or engagement rather than the independent effect of gaming.

The other four studies indicated that exergaming and traditional exercise may both influence BDNF, but superiority was inconsistent (12, 23, 31, 32). A 12-week balance training study reported serum BDNF increases in both exergaming and traditional balance groups, with statistical significance only in the traditional balance group (23). A 3-month community-based trial reported greater BDNF increases after cybercycling than traditional cycling (12), whereas two studies found no significant between-group differences between exergaming and conventional exercise (31, 32). Overall, these findings support peripheral BDNF as a relevant but heterogeneous marker, not as definitive evidence that exergaming is biologically superior to traditional exercise. Thus, similar BDNF responses across exergaming and exercise conditions do not by themselves indicate added neurobiological benefit from game elements.

## 4 Discussion

This scoping review mapped evidence on exergaming and brain-related markers in older adults across neuroelectrical, functional, structural, molecular, and behavioral levels. Overall, many included studies reported favorable within-group changes after exergaming, including modulation of ERP components and EEG rhythms, altered prefrontal activation or resting-state connectivity, selected increases or preservation of brain volume, and increases in peripheral BDNF. However, the evidence does not yet establish that exergaming produces a true cognitive-motor synergistic effect beyond matched physical exercise. Several studies showed effects comparable to traditional exercise, and only a subset reported advantages favoring exergaming. Therefore, our interpretation has been revised from a causal or synergistic conclusion to a more cautious evidence map: exergaming is a promising, engaging form of motor-cognitive training, but its unique active ingredients remain to be isolated. Equivalence to conventional exercise should not be interpreted as incremental neurobiological benefit from the gaming component. It may instead indicate that game-based delivery preserves exercise benefits while potentially improving motivation, enjoyment, adherence, and sustained participation.

The term brain health was also refined in this revision. The included outcomes should not be interpreted as interchangeable indicators of a single construct. Peripheral BDNF reflects a molecular marker related to neurotrophic signaling, MRI volume reflects structural adaptation over longer timescales, fNIRS/fMRI measures reflect task-related or resting-state functional organization, and EEG/ERP measures reflect fast neuroelectrical processing. These levels may be complementary, but they cannot be arranged into a confirmed causal sequence based on the present evidence. The revised synthesis therefore treats them as distinct levels of adaptation that may converge on cognitive and motor behavior rather than as equivalent proof of global brain health improvement.

Reduced prefrontal activation after exergaming deserves particularly cautious interpretation. In some studies, lower prefrontal oxygenation or activation during walking or complex movement tasks may be consistent with improved neural efficiency, especially when accompanied by stable or improved behavioral performance. However, in movement-related paradigms, the same pattern may also reflect greater motor automaticity, reduced reliance on conscious executive control, or task familiarization. These explanations are not mutually exclusive, but they imply different mechanisms. Consequently, decreased activation should be interpreted in relation to the task, behavioral performance, and comparator condition rather than being treated as direct evidence of improved cognitive efficiency.

Behavioral findings provide an important anchor for interpreting neurobiological outcomes. Studies that reported cognitive outcomes generally showed improvement after exergaming, particularly in executive function, attention, or memory-related tasks. Nevertheless, because many studies had small samples, short follow-up periods, and comparators that did not fully match exercise dose, cognitive load, novelty, or enjoyment, the present evidence cannot determine whether improvements resulted from physical exertion, cognitive engagement, game-based motivation, repeated task practice, or their combination. Future studies should use designs that can separate these components, such as factorial comparisons of exercise-only, cognitive-only, combined motor-cognitive training, and exergaming conditions. Therefore, when neurobiological outcomes are comparable, exergaming may still be valuable as a behavioral or adherence-related strategy, even if incremental brain mechanisms are not demonstrated.

A plausible mechanistic model is that exergaming may engage molecular, functional, structural, and behavioral adaptation through repeated motor-cognitive demands. Physical activity may contribute to neurotrophic and vascular responses, including BDNF upregulation, while game-based demands may direct attention, visuospatial processing, decision-making, and feedback learning toward specific neural circuits (4, 5). However, this model remains inferential. The reviewed studies measured different mechanisms in different samples and rarely assessed molecular, structural, functional, and behavioral outcomes simultaneously. Therefore, the proposed pathway from BDNF to structural plasticity to functional efficiency to cognition should be presented as a framework for future longitudinal and mediation-based research rather than as a demonstrated causal chain. This model should be read as a hypothesis-generating framework rather than as evidence that the game layer itself has been shown to drive additional neuroplastic change.

Future mechanistic studies should combine matched active control groups with multimodal assessments and adequate follow-up. Ideally, they should measure exercise intensity, cognitive load, enjoyment, adherence, neurotrophic markers, brain activation/connectivity, brain structure, and cognition in the same participants. Such designs would clarify whether exergaming has unique effects beyond traditional exercise and whether changes in BDNF, brain structure, and functional activation mediate cognitive outcomes. To test a gaming-specific mechanism, future trials should ensure that exercise dose is carefully matched and should isolate the incremental contribution of cognitive challenge, feedback, novelty, and motivational design.

## 5 Methodological limitations

The contradictory findings revealed in this review primarily stem from heterogeneity across three dimensions: intervention protocols, population characteristics, and research methodologies. Together, these define the current limitations and boundaries of the evidence.

First, heterogeneity in intervention protocols is a major source of uncertainty. The exergames used in existing studies differ substantially in motor demands (cycling, dance, balance, stepping, boxing), cognitive demands (attention, inhibition, visuospatial navigation, memory updating, decision-making), immersion level, feedback structure, social context, exercise intensity, and dose. These intervention features likely recruit partly different neural circuits. For example, balance games may emphasize sensorimotor integration and postural control, dance games may combine rhythm, sequencing, and visuomotor coordination, and virtual cycling may emphasize aerobic load and spatial navigation. Future reviews and trials should classify exergames by their active motor and cognitive ingredients rather than treating the presence of gaming itself as the critical mechanism. This distinction is important because the gaming component may primarily improve motivation and adherence rather than directly enhance neuroplasticity beyond dose-matched exercise.

Second, population heterogeneity may have influenced the findings. Aging itself is a highly heterogeneous process. Individuals with cognitive risk (e.g., MCI) or neurodegenerative diseases (e.g., PD), whose brains may be in a compensatory or plasticity-ready state, may respond more sensitively to enriched interventions such as exergaming than cognitively healthy older adults. This possibility was suggested by studies reporting hippocampal volume growth. In addition, baseline fitness, genetic background, and other individual factors may modulate biological and behavioral responses to intervention.

Third, limitations in measurement and design constrain causal interpretation. (1) Most studies measured peripheral BDNF, and the relationship between peripheral and central BDNF remains incompletely understood. (2) Most studies used pre-post measurement only and did not capture real-time neural dynamics during exergaming. (3) Some comparators, such as usual care, cannot isolate the independent contributions of exercise, cognition, novelty, enjoyment, and feedback. (4) No protocol for this review was prospectively registered; this has now been acknowledged as a limitation. (5) Because this was designed as a scoping review, no formal risk-of-bias assessment was conducted. This decision is consistent with the mapping purpose of scoping reviews, but it limits the strength of conclusions about intervention effectiveness. (6) Publication bias cannot be ruled out, particularly because the field contains many small exploratory trials that may preferentially report positive findings. In particular, few studies were designed to test incremental benefit by matching exercise dose while manipulating only the cognitive-game or motivational component.

## 6 Conclusion

Current evidence suggests that exergaming is a promising motor-cognitive intervention for older adults and may be associated with favorable changes in neuroelectrical activity, task-related or resting-state brain function, selected structural brain measures, peripheral BDNF, and cognitive performance. However, the evidence base remains preliminary and heterogeneous. Reduced prefrontal activation may indicate neural efficiency, motor automaticity, or task familiarization depending on context, and the proposed pathway linking BDNF, structural plasticity, functional activation, and cognition should be considered a hypothesis rather than a proven mechanism. Future trials should use larger samples, preregistered protocols, better matched active controls, detailed intervention reporting, and multimodal longitudinal assessment to identify the active ingredients and long-term effects of exergaming. Thus, findings of comparability with conventional exercise should not be interpreted as evidence of a unique gaming-specific mechanism. Demonstrating incremental benefit will require dose-matched designs that separate physical exercise effects from game-based cognitive and motivational components.

## Data Availability

The original contributions presented in the study are included in the article/Supplementary material, further inquiries can be directed to the corresponding author.
