# Supplementary material for: Effects of exergaming on brain health in aging: a scoping review of current evidence
Source: Front Public Health. 2026 Aug 10;14:1912065. doi: 10.3389/fpubh.2026.1912065 (PMC13500335; doi:10.3389/fpubh.2026.1912065)
Supplement: Supplementary file 1 [file Table_1.DOCX]

**Supplementary Table S1. Database-specific search strategies**

Search date: October 20, 2025. The syntax was adapted to the indexing and field-tag conventions of each database.

| **Database** | **Search string** |
| --- | --- |
| PubMed | ((exergam*[Title/Abstract] OR "active video game*"[Title/Abstract] OR "video game*"[Title/Abstract] OR "computer game*"[Title/Abstract] OR "serious game*"[Title/Abstract] OR "virtual reality"[Title/Abstract] OR "virtual environment*"[Title/Abstract] OR "virtual cycling"[Title/Abstract] OR Wii[Title/Abstract] OR Kinect[Title/Abstract]) AND ("Aged"[Mesh] OR "older adult*"[Title/Abstract] OR elderly[Title/Abstract] OR aging[Title/Abstract] OR ageing[Title/Abstract] OR geriatric*[Title/Abstract]) AND (brain[Title/Abstract] OR cognition[Title/Abstract] OR cognitive[Title/Abstract] OR EEG[Title/Abstract] OR ERP[Title/Abstract] OR fNIRS[Title/Abstract] OR fMRI[Title/Abstract] OR MRI[Title/Abstract] OR "magnetic resonance imaging"[Title/Abstract] OR BDNF[Title/Abstract] OR "brain-derived neurotrophic factor"[Title/Abstract])) |
| Scopus | TITLE-ABS-KEY ( exergam* OR "active video game*" OR "video game*" OR "computer game*" OR "serious game*" OR "virtual reality" OR "virtual environment*" OR "virtual cycling" OR wii OR kinect ) AND TITLE-ABS-KEY ( "older adult*" OR elderly OR aging OR ageing OR geriatric* ) AND TITLE-ABS-KEY ( brain OR cognition OR cognitive OR eeg OR erp OR fnirs OR fmri OR mri OR "magnetic resonance imaging" OR bdnf OR "brain-derived neurotrophic factor" ) |
| Web of Science | TS=(exergam* OR "active video game*" OR "video game*" OR "computer game*" OR "serious game*" OR "virtual reality" OR "virtual environment*" OR "virtual cycling" OR Wii OR Kinect) AND TS=("older adult*" OR elderly OR aging OR ageing OR geriatric*) AND TS=(brain OR cognition OR cognitive OR EEG OR ERP OR fNIRS OR fMRI OR MRI OR "magnetic resonance imaging" OR BDNF OR "brain-derived neurotrophic factor") |
| PsycINFO | (TI,AB(exergam* OR "active video game*" OR "video game*" OR "computer game*" OR "serious game*" OR "virtual reality" OR "virtual environment*" OR "virtual cycling" OR Wii OR Kinect)) AND (TI,AB("older adult*" OR elderly OR aging OR ageing OR geriatric*)) AND (TI,AB(brain OR cognition OR cognitive OR EEG OR ERP OR fNIRS OR fMRI OR MRI OR "magnetic resonance imaging" OR BDNF OR "brain-derived neurotrophic factor")) |
